# Low Concentrations of Methamphetamine Can Protect Dopaminergic Cells against a Larger Oxidative Stress Injury: Mechanistic Study

**DOI:** 10.1371/journal.pone.0024722

**Published:** 2011-10-12

**Authors:** Amina El Ayadi, Michael J. Zigmond

**Affiliations:** 1 Pittsburgh Institute for Neurodegenerative Diseases, University of Pittsburgh, Pittsburgh, Pennsylvania, United States of America; 2 Department of Neuroscience and Cell Biology, University of Texas Medical Branch, Galveston, Texas, United States of America; National Institutes of Health, United States of America

## Abstract

Mild stress can protect against a larger insult, a phenomenon termed preconditioning or tolerance. To determine if a low intensity stressor could also protect cells against intense oxidative stress in a model of dopamine deficiency associated with Parkinson disease, we used methamphetamine to provide a mild, preconditioning stress, 6-hydroxydopamine (6-OHDA) as a source of potentially toxic oxidative stress, and MN9D cells as a model of dopamine neurons. We observed that prior exposure to subtoxic concentrations of methamphetamine protected these cells against 6-OHDA toxicity, whereas higher concentrations of methamphetamine exacerbated it. The protection by methamphetamine was accompanied by decreased uptake of both [^3^H] dopamine and 6-OHDA into the cells, which may have accounted for some of the apparent protection. However, a number of other effects of methamphetamine exposure suggest that the drug also affected basic cellular survival mechanisms. First, although methamphetamine preconditioning decreased basal pERK1/2 and pAkt levels, it enhanced the 6-OHDA-induced increase in these phosphokinases. Second, the apparent increase in pERK1/2 activity was accompanied by increased pMEK1/2 levels and decreased activity of protein phosphatase 2. Third, methamphetamine upregulated the pro-survival protein Bcl-2. Our results suggest that exposure to low concentrations of methamphetamine cause a number of changes in dopamine cells, some of which result in a decrease in their vulnerability to subsequent oxidative stress. These observations may provide insights into the development of new therapies for prevention or treatment of PD.

## Introduction

Exposure of cells to subtoxic amounts of stress can provide protection against normally toxic stress levels. This phenomenon, referred to as preconditioning or tolerance, has been studied primarily in models of ischemia [see [1], [2] for reviews]. However, related phenomena have been observed using a variety of stimuli, including anesthetic agents [3], exercise [4],[5], dietary restriction [6], hypothermia and hyperthermia [7], and low doses of toxins [8],[9].

We are particularly interested in determinants of dopamine (DA) neuron death. Loss of DA neurons projecting from the substantia nigra to the striatum is a hallmark of Parkinson's disease (PD). Although the cause of this neurodegeneration is unknown, evidence suggests that oxidative stress, mitochondrial dysfunction, and accumulation of misfolded proteins are involved [10], [11], [12], [13]. Moreover, as in the case of ischemia, several observations suggest that mild stress can be protective in models of PD. Thus, for example, studies in animal models of the DA deficiency associated with PD have indicated protective effects of dietary restriction [14] thrombin preconditioning [15], exercise, forced motor use, and environmental complexity [4], [16], [17].

In vitro studies further support the hypothesis that mild cellular stress can protect cell lines exhibiting some aspects of the DA phenotype. For example, pinacidil, xanthine/xanthine oxidase, and FeSO_4_ have each been shown to protect PC12 cells against concentrations of MPTP or rotenone, which would otherwise kill the cells via inhibition of mitochondrial respiration [18], [19], [20]. Our group has reported that although 6-hydroxydopamine (6-OHDA) is normally toxic to DA cells due to the formation of reactive oxygen species (ROS) [20], exposure of the dopaminergic cell line MN9D, which exhibits many dopaminergic properties [21], to low concentrations of the neurotoxin protects against higher concentrations [20]. Likewise, exposure of PC12 cells to low concentrations of the proteasome inhibitor MG132 can protect against a much larger insult [9].

In this report, we have chosen to study the impact of a single 24-hr exposure of a stressor on the vulnerability of MN9D cells to subsequent intense oxidative stress. For the conditioning stimulus we have used methamphetamine (METH), which is toxic to DA cells at least in part due to the formation of ROS [see reviews by [22], [23], [24]. The challenge stress was 6-OHDA [25],[26]. Our results show that 24 hr exposure to low concentrations of METH protected the cells against subsequent exposure to 6-OHDA. This was associated with an increase in activated extracellular regulated kinase (ERK) and an upregulation of the pro-survival protein Bcl-2. Our results extend the evidence suggesting that exposure to mild oxidative stress induces an upregulation of pro-survival molecules and decreases vulnerability to a larger insult of the same or a different nature.

## Methods

### Materials

Unless otherwise noted, all reagents were obtained from Sigma-Aldrich Corporation, St. Louis, MO and were of the highest available purity.

### Cell Culture

The dopaminergic cell line MN9D was originally obtained from Drs. Alfred Heller and Lisa Won (University of Chicago, Chicago, IL) and are now available from our laboratory at the University of Pittsburgh by arrangement with the University of Chicago. The cells result from a fusion of rostral mesencephalic neurons from embryonic C57BL/6J (E14) mice with the N18TG2 neuroblastoma cells [21]. There are a number of important similarities between these cells and DA neurons [21], and we have found them be a valuable model for studies of the DA deficiency associated with PD [20], [27], [28], [29], as have many others [e.g., [30],[31], [32], [33].

The cells were used in their undifferentiated form between passage 8 and 20 and were plated in Primaria 10 cm plates (BD-Falcon Biosciences, Bedford, MA) in Dulbecco's Modified Eagle's Media (DMEM) (Gibco, Invitrogen Corp, Carlsbad, CA). The media was supplemented with sodium bicarbonate and the pH adjusted to 7.2 before adding 50 units/ml penicillin, 50 mg/ml streptomycin, and, 10% fetal bovine serum (Hyclone, Logan, UT). The cells were kept in an incubator at 37°C with 5% CO_2_.

### Treatments

For viability experiments, MN9D cells were plated at 10,000 cells per well in triplicate in 96-well Primaria plates (BD, Franklin Lakes, NJ), and all treatments were performed 24 hr after plating. Unless otherwise noted, cells were incubated with METH (0–3 mM) or vehicle (standard medium) for 6 or 24 hr in serum-containing medium. The medium was then changed, and the cells were washed with standard medium and treated with 100 µM 6-OHDA for 20 min in a special medium as described below to circumvent its degradation during short term treatments as confirmed by HPLC. The 6-OHDA-containing medium was then removed and the cells were incubated in fresh standard medium for an additional 24 hr until assay. To avoid 6-OHDA breakdown and formation of oxidation products, the drug was made in a vehicle containing 0.15% ascorbic acid and the metal chelator diethylenetriamine penta-acetic acid (DETAPAC, 10 mM) and flushed for 10 min with nitrogen gas to further reduce oxidative degradation of 6-OHDA [34]. Lighting conditions were also kept minimal during treatment for the same reason. The effects of 6-OHDA were always compared with the effects of its vehicle. When the MAP kinase kinase (MEK1/2) inhibitor U0126 (Calbiochem, EMD Chemicals Inc. San Diego, CA) was used, cells were pretreated for 1 hr with 10 µM U0126 or its vehicle before and during the time of 6-OHDA exposure. Because very little toxicity was seen 6 hr post-METH treatments (data not shown) we chose to use the 24 hr time point, which is the most common time point used for in vitro studies looking at the effect of METH on cell viability [35], [36], [37], [38], [39].

### WST-1 assay

We used the WST-1 cell proliferation assay (Roche Applied Science, Indianapolis, IN) to estimate the number of viable cells by detecting the cleavage of tetrazolium salts by the mitochondrial dehydrogenases, which is affected by the availability of NADH, the redox status of the cell, and the presence of ROS. MN9D cells were treated as described above, and 23 hr after treatment with 6-OHDA the medium was removed and replaced with a fresh medium containing the WST-1 reagent. Absorbance at 440 nm was measured using an ELISA plate reader after 30 min incubation at 37°C.

### CellTiter-Glo assay

Another non-biased viability index used was the Cell Titer-Glo luminescent assay. This assay is based on a luciferase/luciferin reaction that, in the presence of Mg^2+^ and ATP, produces oxyluciferin and releases energy in the form of luminescence. Since the luciferase reaction requires ATP, the luminescence produced is proportional to the amount of ATP present, an indicator of cellular metabolic activity. We used the Cell Titer-Glo kit (Promega, MA) according to the manufacturer instructions. Briefly, the plate was taken from the incubator and brought to room temperature, and 100 µl of Cell Titer-Glo mixture were added to each well. The cells were then lysed by shaking the plate at high speed for 10 min. After 10 min, we transferred 100 µl from each well to another 96-well plate. The luminescence reading was performed using a Vector 96-well plate reader (Perkin-Elmer, Boston, MA).

### Hoechst reagent assay

We used bisbenzimide (Hoechst 33258) to quantify nuclear condensation in cells going into apoptosis. This reagent is relatively selective for double stranded DNA and binds specifically to the A-T base pairs in the DNA, resulting in an increase in fluorescence. Twenty-four hr after 6-OHDA treatment, the medium was removed, and the cells fixed using 100 µl of 4% paraformaldehyde solution in 4% sucrose for 20 min. The fixation solution was removed and replaced with 100 µl staining solution (1 µg/ml Hoechst reagent) for another 20 min. The staining solution was then taken out and replaced with 100 µl PBS with 0.01% azide and the cells were then stored at 4°C until analysis. Photomicrographs of each well were taken at 20× using MetaMorph software (Molecular Devices, Downingtown, PA) with a Nikon Inverted Eclipse microscope and the number of viable cells counted while being blind to the treatment.

### ^3^H-DA uptake assay

The ^3^H-DA uptake assay was adapted from defazio et al., [40] with some modifications. MN9D cells were treated with METH for 24 hr. The media was then aspirated and the cells incubated with 50 nM ^3^H-DA in PBS supplemented with 25 mM glucose, 1 mM CaCl_2_, and 1 mM MgCl_2_ for 15 min at 37°C. The specific activity of this batch of ^3^H-DA was 59.3 Ci/mmol (American Radiolabeled Chemicals, St. Louis, MO). Following 3 washes with ice-cold supplemented PBS, ^3^H-DA was extracted from culture twice using 0.1 N perchloric acid and 25% ethanol and added to Cytoscint scintillation fluid (ICN, Costa Mesa, CA). The DA uptake was measured using a liquid scintillation spectroscopy (Beckman LS-1800; Beckman Coulter, Inc. Fullerton, CA). Non-specific uptake was determined by adding the DA uptake inhibitor nomifensine (10 nM) prior to and during the assay. In another set of experiments, cells were treated with METH for 24 hr, the media was changed and the cells incubated for 20 min with vehicle alone or vehicle plus 6-OHDA before performing the DA uptake assay as described above.

### Assay of 6-OHDA

HPLC coupled with electrochemical detection was used to measure the amount of 6-OHDA taken up into MN9D cells using minor modifications of previous methods [41], [42]. After the incubation was complete, the cells were collected by centrifugation, washed, lysed in 0.1 N HClO_4_ containing 0.2 mM NaHSO_3_, and an aliquot injected onto a ESA C18 column (2.1×150 mm, ESA, Inc., Chelmsford, MA). The mobile phase was pumped through the system at 0.3 ml/min using a ESA LC-10AD pump (ESA Inc., Chelmsford, MA). Analytes were detected coulometrically using an ESA Coulochem Model 4100A detector, an ESA Model 5010 conditioning cell, and an ESA Model 5014B microdialysis cell (ESA Inc., Chelmsford, MA).

### Western Blot analysis

MN9D cells were plated in 6-well plates at the density of 500,000 cells per well and lysates were collected at different times points after treatment. Cells were washed with sterile chilled Dulbecco's phosphate buffer saline (DPBS) and then lysed with a 1% Triton X-100 lysis buffer containing 20 mM Tris (pH 6.8), 137 mM NaCl, 25 mM beta glycerophosphate, 2 mM NaPPi, 2 mM EDTA, 1 mM Na_3_VO_4_, 10% glycerol, 5 µg/ml leupeptin, 5 µg/ml aprotinin, 2 mM benzamidine, 0.5 mM DTT, and 1 mM PMSF. Cells were collected in ice cold lysis buffer and lysed for 30 min before centrifugation at 4°C for 30 min at 15 000 g. Protein content was determined using the bicinchoninic acid (BCA) assay (Pierce, Rockford, IL), and 20 µg protein from each sample was separated on a 10% SDS gel and transferred to a PVDF membrane (Bio-Rad labs, Hercules, CA). The blots were then blocked in 5% non-fat milk and incubated overnight at 4°C with primary antibodies. After washes, membranes were incubated at room temperature for 60 min with diluted horseradish peroxidase-conjugated anti-rabbit secondary antibody (1∶10000; Calbiochem, San Diego, CA). Bound antibody was visualized by chemiluminescence (NEN, Boston, MA), and the blots were then stripped and re-probed for total protein. Equal protein loading was confirmed by probing for α-tubulin. Polyclonal antibodies were purchased from cell signaling (Cell Signaling Technology, Danvers, MA) and used at the following concentrations; pERK1/2 (Thr202/Tyr204, Cat. no. 9101) at 1∶1000; pMEK1/2 (Ser217/221, Cat. no. 9121) at 1∶1000; pAkt (Ser473, Cat. no 9271) at 1∶200; pJNK (Thr183/Tyr185, Cat. no. 9251) at 1∶200; total ERK1/2 at 1∶1000 (Cat. no. 9102); total MEK1/2 at 1∶1000 (Cat. no. 9122); total Akt at 1∶1000 (Cat. no 9271) and total JNK at 1∶200 (Cat. no. 9252). Antibodies for CuZnSOD and MnSOD were from Upstate (Lake placid, NY) and were used at the following concentrations: CuZnSOD at 1∶250 (Cat. no. 07–403), MnSOD at 1∶600 (Cat. no. 06–984). Polyclonal rabbit anti-Bcl-2 was used at 1∶200 (Cat. no. sc-4096 WB; Santa Cruz Biotechnology; Santa Cruz, CA).

### Protein phosphatase activity assay

Controls and cells treated with sub-lethal concentration of METH were lysed in a lysis buffer containing 2 mM EDTA, 2 mM EGTA, and 20 mM Imidazole-HCl, pH 7.0. The lysis buffer was supplemented with proteases inhibitors but not with phosphatases inhibitors. The protein phosphatase 2 (PP2A) immunoprecipitation and activity assays were performed using a non-radioactive PP2A activity assay kit (Upstate Biotechnologies, Lake Placid, NY). Following the manufacturer instructions, 500 µg protein from each sample were incubated for 2 hr at 4°C with the PP2A antibody 16D specific to the catalytic subunit (PP2Ac) (Upstate Biotechnologies). The protein A-Sepharose beads (2 µg) were then added to the lysates and the incubation continued overnight. Cells were then washed with the Ser/Thr buffer and equivalent immunoprecipitation of PP2A in all samples was confirmed by immunoblot with PP2Ac. Immunoprecipitated PP2A was then tested for activity measuring dephosphorylation of the phosphopeptide KRpTIRR spectrophotometrically at 650 nm using the malachite green [43].

### Statistical analysis

Statistical significance was determined using ANOVA (Graph Pad, San Diego, CA) and the Student-t test for statistical comparisons between groups. Each experimental condition was repeated in triplicate on at least 3 separate occasions. Data comparisons were considered significant if the *p-*value was 0.05 or less.

## Results

### 6-OHDA and METH caused cell death in concentration-dependent manners

**Figure 1:**
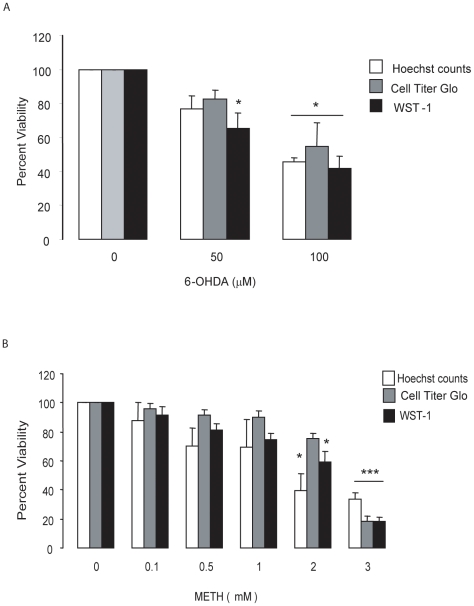
METH and 6-OHDA induced toxicity in MN9D cells. (A) MN9D cells were treated with 6-OHDA for 20 min and viability assays performed 24 hr after 6-OHDA removal. 6-OHDA killed MN9D cells in a concentration-dependent fashion with an EC_50_ of approximately 100 µM. (B) MN9D Cells were treated with the indicated concentrations of METH for 24 hr and viability assays performed 24 hr later. METH affected ATP levels, mitochondrial dehydrogenase activity, and chromatin condensation as assessed by Hoechst staining. The average EC_50_ for METH for the three different viability assays was between 2 and 3 mM. Data represent means ± SEM of 3–5 independent experiments. *P<0.05, ***p<0.001 compared to controls.

MN9D cell were exposed to medium containing 6-OHDA for 20 min, which was then replaced with standard growth medium, and cell viability was assayed 24 hr later. We observed that 6-OHDA decreased cell viability in a concentration-dependent manner. At 100 µM, 6-OHDA reduced cell viability by approximately 50% when assayed by Hoechst staining followed by cell counts based on morphological change such as nuclear condensation and staining intensity. At 500 µM 6-OHDA, MN9D cell viability was reduced by 90% (data not shown) confirming previous findings from our lab [28], [29]. In addition, an EC_50_ comparable to that obtained with the Hoechst reagent was obtained with the Cell Titer-Glo assay, which estimates cell viability by measuring changes in ATP levels, and the WST-1 assay, which estimates cell density by measuring changes in NADH activity (Fig. 1A). For all subsequent experiments we used 100 µM 6-OHDA in order to detect changes in cell viability when pre-treating with METH.

Next, cells were exposed to METH for 24 hr and the decrease in cell viability after an additional 24 hr was assessed. We observed that the drug decreased basal cell viability in a concentration-dependent manner with an EC_50_ between 2 and 3 mM, depending on the assay used (Fig. 1B). We did not see any significant effect of METH concentrations on cell viability at or below 1 mM with any of the three viability assays used. From these observations, we selected 0.5 and 1 mM METH as pretreatment concentrations for preconditioning studies.

### Pretreatment with low concentrations of METH protected cells against 6-OHDA toxicity

**Figure 2:**
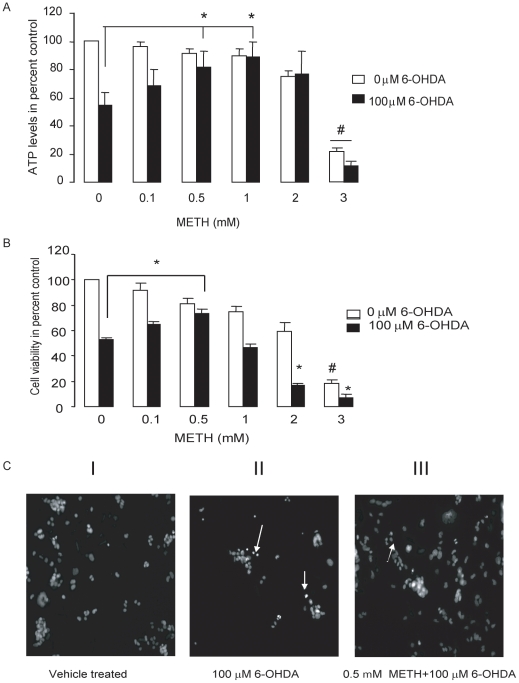
Sub-lethal concentrations of METH protected MN9D cells against 6-OHDA toxicity. Cells were treated with the indicated concentrations of METH for 24 hr. The medium was then changed, the cells treated with 100 µM 6-OHDA for 20 min and viability assays performed 24 hr after 6-OHDA removal. Sub-toxic concentrations of METH protected ATP levels (A) and mitochondrial dehydrogenase activity (B), and prevented chromatin condensation of Hoechst stained cells (C). The photomicrographs in Panel (C-I) show control cells lacking any sign of nuclear condensation or chromatin clumping, In panel (C-II) MN9D cells have lifted off the plate after 6-OHDA exposure, presumably due to cell death. Arrows point to dying cells with high chromatin condensation. In panel (C-III) METH pretreated cells were protected against 6-OHDA toxicity (C-III). Data represent means ± SEM (N = 3). *P<0.05 compared to 0 mM METH/100 µM 6-OHDA, #p<0.01compared to 0 mM METH/0 µM 6-OHDA.

MN9D cells were treated with METH for 24 hr. The METH-containing medium was then removed and the cells exposed to medium containing 6-OHDA for 20 min, after which it was replaced with standard growth medium, and cell viability was measured 24 hr later. As we had previously shown (Fig. 1B), exposure of MN9D cells to 0.5 or 1.0 mM METH had no significant effect on their viability as assessed by Cell Titer-Glo or WST-1 assays. However, prior exposure of cells to these concentrations of METH greatly reduced 6-OHDA-induced cell death as measured by each of these methods (Fig. 2A, B, and C). In contrast, no protection was seen with any of our assays when we used higher concentrations of METH that were themselves toxic (e.g., 3 mM). Indeed, the toxic effects of 6-OHDA were increased by prior exposure to these high concentrations of METH (Fig. 2A and 2B). Morphological changes induced by 6-OHDA included shrinkage of the cytoplasmic membrane and chromatin condensation, which are consistent with an apoptotic form of cell death (Fig. 2C).

### METH inhibited the uptake of [^3^H] DA and the accumulation of 6-OHDA

METH is known to reversibly interfere with the high affinity DA transporter [44], [45], [46] Thus, one explanation for the METH-induced tolerance towards 6-OHDA toxicity was that the drug reduced the uptake of 6-OHDA into the MN9D cells. Several experiments were performed to examine this possibility.

**Figure 3:**
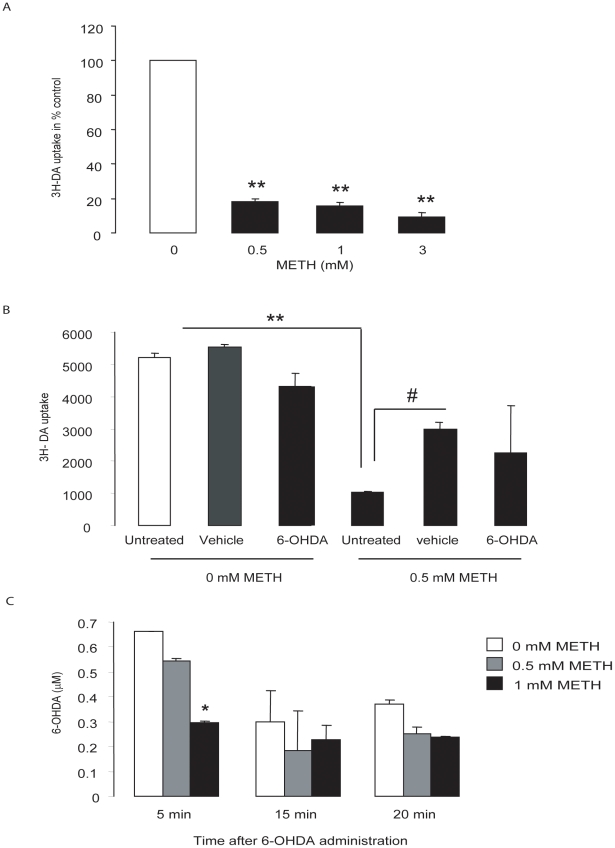
METH affects dopamine transporter function. (A) MN9D cells were treated with different concentration of METH for 24 hr and ^3^H-DA uptake assayed with METH still in the media as described in the methods section. (B) MN9D cells were treated with METH for 24 hr, the media was changed, the cells were then treated with vehicle or 6-OHDA (100 µM) for 20 min, and DA uptake assayed immediately after. (C) MN9D cells were treated with METH for 24 hr, the media was changed and cells treated with 100 µM 6-OHDA or vehicle. The cells were collected at different time points to analyze 6-OHDA content using HPLC-EC. Data are presented as means ± SEM (N = 3). *p<0.05, **p<0.01 compared to 0 mM METH, #p<0.05 compared to 0.5 mM METH.

First, we measured ^3^H-DA uptake after 24 hr of exposure to 0.5 mM METH and then washed the cells with standard medium without METH before incubation for 20 min with 6-OHDA, thus precisely mimicking the protocol for our preconditioning experiments. Under these conditions, METH-induced inhibition of DA uptake was reduced to 38% (Fig. 3B), a sizable effect but a significantly less than the impact of the same concentration of METH on viability (Fig. 2). In contrast, 24 hr of incubation with 0.5 mM METH and *without* a wash before 6-OHDA decreased DA uptake by 75% to 80% (p<0.01) (Fig. 3A).

Next, we measured the amount of 6-OHDA that accumulated in MN9D cells pre-treated with 0.5 or 1 mM METH for 24 hr using HPLC. We observed that METH reduced the accumulation of 6-OHDA in a concentration- and time-dependent manner. When cells were exposed to 0.5 or 1 mM METH, no significant difference in 6-OHDA accumulation was observed at either 15 or 20 min, the time points at which we would normally stop 6-OHDA treatment in our preconditioning experiments. In contrast, after only 5 min of exposure to 6-OHDA, 1 mM (but not 0.5 mM) METH reduced 6-OHDA accumulation by 55% (p<0.05) when cells were preconditioned (Fig. 3C). Collectively, these results suggest that METH pretreatment may have reduced 6-OHDA toxicity in MN9D cells in part by an initial reduction in 6-OHDA uptake, but that this reduction was relatively small and transient, and thus not able to explain the preconditioning we observed. That assumption was further supported by the additional experiments that we will now describe.

### METH decreased pERK1/2, but potentiated the subsequent pERK1/2 response to 6-OHDA

**Figure 4:**
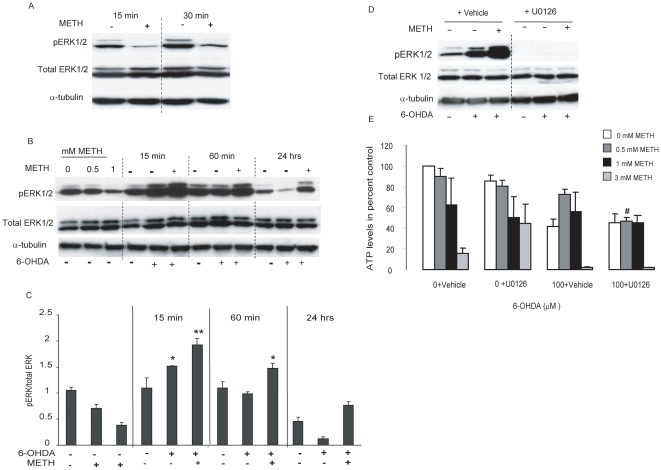
METH decreased basal pERK1/2 levels and potentiated ERK1/2 phosphorylation induced by 6-OHDA. (A) Western blot analysis showing pERK levels at different time points after exposure to sub-lethal concentration of METH (0.5 mM) for up to 24 hr. Lysates were collected at 15 min, 30 min, 1 hr, 6 hr, and 24 hr. Shown are the 15 min and 30 min time points. (B) pERK1/2 levels decreased by 15 min post METH exposure and lasted for up to 24 hr. METH decreased activation of ERK 1/2 in a concentration-dependent manner (0, 0.5, and 1 mM at the 24 hr time point) and enhanced the activation of ERK1/2 after 6-OHDA exposure. This activation lasted from 15 to at least 24 hr. (C) Quantification of ERK1/2 activation in response to 6-OHDA. (D) MN9D cells were treated with sub-lethal concentration of METH (0.5 mM) for 24 hr, the medium was removed and the cells treated with the MEK1/2 inhibitor U0126 (10 µM) for 1 hr before and during the 20 min 6-OHDA exposure. Western blot analysis confirmed the activation of ERK1/2 by 6-OHDA and its enhancement by METH preconditioning and confirmed that U0126 blocked the phosphorylation of ERK1/2. (E) Viability assay looking at ATP levels and performed 24 hr post 6-OHDA treatment showed that METH protected MN9D cells against 6-OHDA toxicity and U0126 abolishes this protection. α-tubulin was used as a loading control. Data are presented as means ± SEM (N = 3). *p<0.05, **p<0.01 compared to vehicle and ^#^p<0.05 compared to lane 10.

To determine if METH also produced intracellular signaling changes that might contribute to its ability to protect cells against 6-OHDA, we looked at ERK, a member of the MAP kinase family that has frequently been shown to promote cell survival. Changes in the levels of the activated, phosphorylated form of ERK1/2 were determined by Western blot analysis using a phospho-specific ERK1/2 antibody. A low concentration of METH (0.5 mM) by itself caused a marked decline in pERK1/2 that was prominent after 15 min (Fig. 4A) and still detectable 24 hr after treatment (Fig. 4B). Treatment with 6-OHDA alone induced a biphasic increase in pERK1/2 that peaked at 15 min, returned to basal levels by 1 hr, and subsequently decreased below basal levels by 24 hr (Fig. 4B, C). However, despite the possibility that METH pretreatment may have decreased the uptake of 6-OHDA into the MN9D cells (see above), pre-incubation with METH actually caused a persistent *increase* in the levels of pERK1/2 normally seen after 6-OHDA exposure (Fig. 4B, C). These same effects were seen at all time points examined.

### Inhibition of ERK activation reduced the protective effect of METH

MEK 1/2 is the upstream activator of ERK1/2. Thus, we used the MEK1/2 inhibitor, U0126, to determine if the METH-induced changes in pERK1/2 were related to the METH-induced protection against 6-OHDA. Addition of U0126 (10 µM) for 1 hr before and during exposure to 6-OHDA or its vehicle, inhibited the activation of pERK1/2 due to METH and 6-OHDA treatments with no change in total ERK1/2 protein (Fig. 4D). It also reduced the toxic effects of high concentrations of METH, though not of 6-OHDA (Fig. 4E). Nevertheless, U0126 abolished the protection against 6-OHDA-induced cell death that was provided by METH pretreatment. Those effects were not seen when the cells were treated with the U0126 vehicle.

### Low concentration of METH increased MEK1/2 activation in response to 6-OHDA

**Figure 5:**
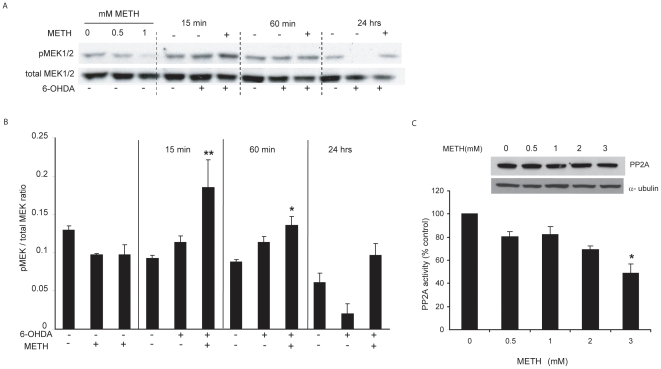
METH preconditioning affected MEK1/2 and PP2A. (A) A representative blot showing that sublethal concentrations of METH decreased the levels of pMEK1/2 and enhanced MEK1/2 activation induced by 6-OHDA. MN9D cells were treated with METH for 24 hr, the medium was changed, and cells treated with 6-OHDA for 20 min. Lysates were collected at 0, 15 min, 60 min, and 24 hr post 6-OHDA treatment. (B) Quantification of panel A in 4 independent experiments. (C) PP2A activity assay was performed as described in the methods section from cells treated with various METH concentrations for 24 hr. METH pretreatment decreased PP2A activity in a concentration dependent manner, although total cellular PP2A levels were not affected. *p<0.05, **p<0.01 compared to control.

The previous experiment showed that inhibition of pERK1/2 activation blocked the protection by METH against 6-OHDA. This suggested that pERK1/2 was somehow involved in METH-induced preconditioning. To examine this further, we looked at the effect of METH on MEK1/2. We observed that pMEK1/2, like pERK1/2, was decreased by pretreatment with a low concentration of METH (0.5 mM) (Fig. 5A). However, again as in the case of pERK1/2, MEK1/2 phosphorylation was increased by 6-OHDA treatment and this effect was potentiated by METH preconditioning (Fig. 5A, B).

### METH preconditioning decreased PP2A activity

The levels of pERK are determined by the opposing effects of two phosphoproteins, pMEK, which increases pERK, and PP2A, which decreases it. To determine PP2A activity after METH preconditioning, we used a two-step assay comprised of PP2A immunoprecipitation followed by quantification of its activity [43]. To ensure comparability, we first confirmed that equal amounts of PP2A had been immunoprecipitated in control and METH treated MN9D cells by using an antibody specific to the catalytic subunit 1D6 (PP2Ac), and then measured the effect of METH treatment on PP2A activity. We found that 24 hr exposure to 0.5–3 mM METH had no effect on PP2A protein levels, but decreased PP2A activity in a concentration-dependent fashion.

### METH preconditioning also enhanced the response of pAkt but not pJNK to 6-OHDA

**Figure 6:**
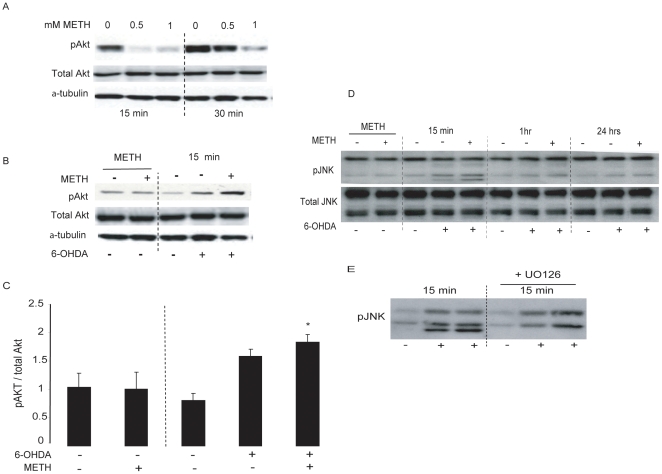
METH preconditioning enhanced Akt but not JNK activation in response to 6-OHDA. MN9D cells were treated with a sub-lethal concentration of METH for 24 hr and then exposed to 100 µM 6-OHDA for 20 min before the cells were lysed at 15 min, 30 min, 1 hr, 6 hr, and 24 hr post 6-OHDA. (A) METH decreased pAkt levels as shown for the 15 and 30 min time points. (B) METH preconditioning potentiates Akt activation in response to 6-OHDA and no effect was seen on total Akt levels. (C) Quantification of pAkt compared to total Akt in 4 independent experiments. (D, E) METH preconditioning has no effect on basal or 6-OHDA induced phosphorylation of JNK. α-tubulin was used to control for protein loading. Data are means ± SEM (N = 3). *p<0.05 compared to control.

The serine/threonine-specific protein kinase Akt is another common intracellular survival factor, and over-expression of activated Akt has been shown to protect against METH toxicity [47]. To ascertain whether the Akt pathway plays a role in METH preconditioning against 6-OHDA toxicity, we examined changes in Akt phosphorylation at residue Ser473, which is required for Akt activity [48] and has been shown to be involved in preconditioning [49]. As in the case of pERK, we found that pAkt levels decreased in response to METH at 15 min and 30 min and returned to baseline levels by 24 hr (Fig. 6A), However, METH pretreatment for 24 hr enhanced Akt phosphorylation seen 15 min after 6-OHDA insult (Fig. 6B,C).

In contrast to ERK and Akt, Jun N-terminal kinase (JNK) is more commonly considered a pro-death signaling molecule. To examine the role of JNK in our experimental paradigm, lysates from control and METH-treated cells were assayed for phosphorylated and total JNK. We observed that 24 hr exposure to a low concentration of METH had no effect on pJNK levels. After 6-OHDA incubation, we saw an activation of pJNK (mainly the 46 kDa immunoreactive band and not the 56 kDa band). However, METH pretreatment did not affect the activation of pJNK1 in response to 6-OHDA (Figure 6D). The immunoblot depicting the effect of METH preconditioning on JNK1/2 activation also revealed the presence of a third immunoreactive band with a molecular weight of approximately 42 kDa. This band is likely to represent an interaction of the JNK antibody used in our experiments with the pERK2 band, which also has a molecular weight of 42 kDa, since this band disappeared when pre-treating the cells with U0126 (Figure 6E).

### METH preconditioning had no effect on the levels of either MnSOD or CuZnSOD, but increased Bcl-2

Previous studies on preconditioning related to ischemia suggest that mild oxidative stress is a perquisite for preconditioning and that antioxidant enzymes are upregulated in response to oxidative stress [9], [18], [50]. As noted above, there is also increasing evidence that METH causes neurotoxicity by increasing the generation of ROS. Based on these findings, we hypothesized that the concentrations of METH that caused preconditioning also caused a mild oxidative stress and that this might have stimulated an counter-regulatory increase in antioxidative capacity; indeed, such an observation would be consistent with *in vivo* findings [51], as well as the findings from our lab in which MG132 was used in conjunction with 6-OHDA in PC12 cells [9].

**Figure 7:**
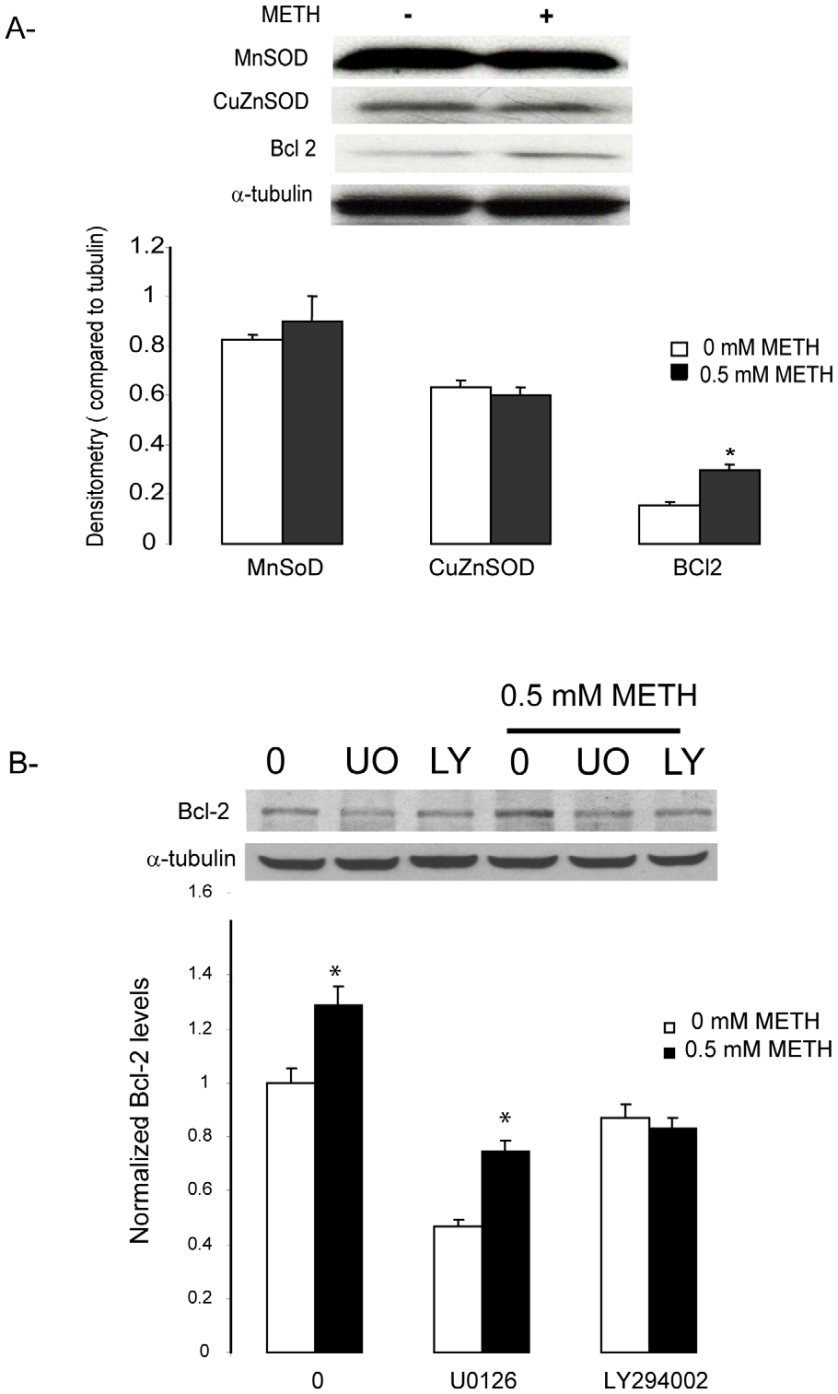
Effect of METH preconditioning on MnSOD, CuZnSOD, and Bcl-2 levels. (A) MN9D cells were treated with 0.5 mM METH for 24 hr and the cells lysates were analyzed for MnSOD, CuZnSOD, and Bcl-2 levels using Western blot analysis. Densitometry analysis showed that METH preconditioning did not affect MnSOD and CuZnSOD but significantly increases Bcl-2 levels. (B) METH increases Bcl-2 in basal conditions and in the presence of the ERK1/2 inhibitor U0126 but has no effects on Bcl-2 levels if the cells are pretreated with the Akt inhibitor LY294002. α-tubulin used as a loading control. Data are means ± ESM (N = 3). *p<0.05 compared to control.

To test our hypothesis, we used Western blot analysis to examine the change in the levels of two key antioxidant enzymes, MnSOD and CuZnSOD after METH preconditioning. However, we observed that 24 hr after METH treatment (0.5 mM), there was no change in the levels of either MnSOD or CuZnSOD (Fig. 7A).

We also examined Bcl-2, an anti-apoptotic protein whose expression is up-regulated in response to mild stress [52], [53]. Bcl-2 has also been found to protect against METH-induced toxicity [54]. After 24 hr exposure to 0.5 mM METH, we observed a significant 28% up-regulation of this protein (Fig. 7B), Inhibition of the ERK and Akt pathways decreases Bcl-2 expression levels in the cells by 55% and 20% respectively. However, Bcl-2 expression level was only decreased by 25% when the cells were preconditioned for 24 hrs with 0.5 mM METH prior to U0126 treatment.

## Discussion

Oxidative stress is widely implicated in neuronal cell death associated with several neurodegenerative disorders, including PD, Alzheimer's disease, and Huntington's disease, as well as cell death following ischemic stroke [11],[55], [56], [57]. In this report, we have examined the effect of METH exposure on the subsequent vulnerability of DA neurons to oxidative stress induced by the DA neurotoxin, 6-OHDA. We used the dopaminergic cell MN9D, which recapitulates many features of DA neurons. We found that high, toxic concentrations of METH intensified the decrease in metabolic activity induced by 6-OHDA as shown by the WST-1 assay. However, we also observed that lower, sub-toxic concentrations of METH attenuated the decline in ATP levels and in mitochondrial dehydrogenase activity, and prevented the nuclear condensation normally seen after exposure to 6-OHDA. This finding is consistent with many previous reports. For example, METH has also been shown to induce a tolerance to the neurotoxic effects of MDMA [58], other amphetamines [59], [60], [61], [62], [63], methamphetamine [64] and MPTP oxidation products [65], [66] as well as to 6-OHDA in an animal model of PD [67]. Moreover, preconditioning and cross-tolerance using several different drugs have been shown in various in vitro models of the DA deficiency seen in PD [19], [20], [53].

### The role of Erk1/2, Akt and JNK in METH preconditioning

ERK1/2 has been shown to be activated in response to oxidative stress and trophic factors in a concentration- and a time-dependent manner and to be involved in both cell death and cell survival [68], [69], [70], [71], [72], [73]. In this study, 6-OHDA increased ERK1/2 phosphorylation as early as 15 min post-exposure. It is generally accepted that the early transient activation of ERK1/2 promotes cell survival by activating anti-apoptotic signaling pathways [29], [74]. We have previously shown that 6-OHDA activates ERK1/2 in a biphasic manner and inhibition of the early ERK1/2 peak exacerbates 6-OHDA toxicity, suggesting that early activation of ERK is a self-protection response to the toxin [30]. In our study METH pre-exposure significantly enhanced the transient ERK1/2 activation in response to 6-OHDA, suggesting that METH preconditioning is partially mediated through the activation of ERK1/2, which would be consistent with many other studies [75], [76], [77], [78], [79]. Such a role for ERK1/2 is further supported by our observation that the MEK1/2 inhibitor U0126 blocked the protective effect of METH preconditioning.

Paradoxically, METH pre-conditioning reduced basal levels of pERK1/2 as well as pMEK1/2, a direct ERK1/2 upstream component. However, we also observed a METH-induced decrease in the activity of PP2A, a protein phosphatase implicated in ERK1/2 dephosphorylation. From these observations, we conclude that METH inhibits both pMEK1/2 and PP2A with the net result depending on the stress level in the cells. In our study, the balance between these two regulators of ERK phosphorylation resulted in a decrease in pERK1/2 under basal conditions but an increase in pERK1/2 during heightened oxidative stress caused by 6-OHDA.

As in the case of ERK1/2, METH preconditioning decreased pAkt levels at early time points while enhancing its response to 6-OHDA. Akt activation has been reported in various preconditioning paradigms [80], [81]. The activation of Akt in our paradigm may reflect two separate events or an inter-dependent activation of the ERK1/2 and PI3K pathways, which have both been shown to be triggered by the activation of the G-protein coupled receptors and growth factors receptors in cells [82].

In addition to its well established role in the execution of apoptosis [83], the JNK pathway is likely to be involved in the neurodegenerative cascade and has been implicated in the loss of DA neurons in PD [for review, see [84], [85]. However, whereas JNK phosphorylation has been reported involved in cell death and survival, the role of JNK in the setting of ischemic preconditioning is controversial with studies suggesting both a protective and detrimental aspects of JNK activation [86], [87]. METH preconditioning had no effect on JNK phosphorylation in response to 6-OHDA in our experiments suggesting that this pro-death pathway is not involved in METH preconditioning.

### Implication of the increase in Bcl-2 in preconditioning in the absence of an increase in SOD isoforms

Antioxidant enzymes isoforms MnSOD and CuZnSOD, as well as heat shock protein, have been implicated in preconditioning in various diseases models [88], and it has been suggested that mild oxidative stress may be a prerequisite for preconditioning [9], [18], [20], [50]. Thus, we predicted that mild oxidative stress would increase SOD levels. However, no changes in either MnSOD or CuZnSOD were detected 24 hr after pretreatment of MN9D cells with low, sub-toxic concentrations of METH. It is possible, of course, that change in one or both of these enzymes does occur earlier and was missed in our single time point analysis, or that other anti-oxidant defenses are stimulated by METH preconditioning.

On the other hand, we did observe a large, significant increase in the anti-apoptotic protein, Bcl-2. This protein has previously been shown to decrease significantly following various toxic regimens of METH [89],[54], [90], and to be involved in various other preconditioning paradigms [20], [52], [53]. Thus, both our own findings and those previously reported by others suggest to us that Bcl-2 up regulation by low levels of METH acts to thwart the activation of pro-apoptotic pathways and to trigger the activation of pro-survival pathways in order to ultimately protect cells against higher levels of oxidative stress. We further suggest that this effect may be independent of the Ras/ERK pathway since Bcl-2 levels were increased in the presence of ERK1/2 inhibitor U0126 in preconditioned cells although the drug reduced basal Bcl-2 levels and in accordance with previous studies [90].

### The significance of METH-induced inhibition of DAT

METH reduced the uptake of DA in MN9D cells, a finding consistent with previous reports as we have noted above. Therefore, it was possible that the uptake of 6-OHDA into MN9D cells is similarly altered, and thus that a component of the apparent protection we observed was due to a decrease in 6-OHDA transport into the cells. Our observations did, indeed, indicate that METH inhibited DAT. However, it is highly unlikely that this alteration of DA transport activity due to METH preconditioning can explain the entire reduction in the vulnerability of the cells to 6-OHDA. First, in our studies of DA uptake that were designed to most closely mimic the conditions of our preconditioning experiments, these effects were both small and transient. Pretreatment with METH reduced 6-OHDA uptake, but only by 20% and even this could only be detected during the initial 5 min of toxin exposure; no effect of METH on 6-OHDA levels was seen at 15 and 20 min, periods during which we have previously shown that 6-OHDA remains in the medium [34]. Second, although 6-OHDA uptake may have been somewhat reduced, 6-OHDA toxicity as measured by cell loss was actually exacerbated by high METH concentrations suggesting that 6-OHDA was still getting into the cells at toxic levels even at the highest METH concentrations. Third, 0.5 and 1 mM METH decreased DA uptake to the same extent, whereas the protection they provided against 6-OHDA toxicity was concentration-dependent. Fourth, many of the effects of 6-OHDA on intracellular signaling molecules, such as the increases in pERK, pAkt, pMEK, were actually intensified by METH pretreatment despite the transient reduction in 6-OHDA uptake. In short, it seems that under our experimental conditions 6-OHDA continues to be taken up into the cells after a very short-lived METH-induced inhibition of this process, which is in accordance with previous data showing a reversible reduction of DA uptake in synaptosomes following pretreatments with DA [91] or METH [92]. Indeed, given the METH-induced reduction in 6-OHDA uptake, it seems likely that all of effects of METH on the response of the MN9D cells to 6-OHDA were actually significantly under-estimated.

### How then might METH reduce the sensitivity of the cells to 6-OHDA?

There is still much to be learned about how exposure to sub-toxic concentrations METH protects MN9D cells against 6-OHDA. However, our results suggest some general outlines of such a mechanism. Bcl-2 exerts a protective effect on cells via its capacity to prevent activation of pre-apoptotic stimuli, and to activate ERK and Akt [92]. The levels of pERK, on the other hand, are regulated by pMEK and PP2A. Both pERK and pMEK are increased by the high levels of ROS generated by 6-OHDA and we hypothesize that METH preconditioning shifts the pMEK/PP2A response to 6-OHDA such that pERK levels are increased and cell survival is thereby favored. This appears also to involve both activation of Akt and an increase in Bcl-2 levels. This general scheme for preconditioning of dopaminergic cells by mild stress is similar to that proposed for other cells, be they from heart [92], [93], kidney [92], [94], or hippocampus [95], [96], in which the stress response has been elicited through mild ischemic episodes. A similar scenario has been suggested for protease activated receptors (PAR) where the activation of PARs is involved in both neurodegeneration and neuroprotection in the brain, depending on the amplitude and the duration of agonist stimulation [97]. PARs are G-protein coupled receptors that regulate cellular response to extracellular serine proteases, like thrombin, trypsin, and trypase. These studies reveal that the MAPK plays a critical role in PAR-mediated neuroprotection suggesting that both cell death and cell survival may share initial signaling proteins, but differences in the amplitude as well as the duration of the signal may result in finally opposite consequences [98]. PAR preconditioning was shown in several animal models of neurodegenerative diseases. For example, it has been shown that thrombin activated PAR-1 can be protective in a rat 6-OHDA model, reducing both neurological deficits and DA terminal loss [15], [99]. Thrombin induced PAR-1 activation was also shown protective against synuclein induced toxicity [100] and to attenuate Aβ-induced cell death of hippocampal neurons and astrocyte stellation [101]. The role of prothrombin/thrombin-induced activation of PARs in neuroprotection and neurodegeneration has been extensively reviewed, and the mechanism by which high concentrations of thrombin exacerbates brain damage while low concentrations of thrombin rescue neural cells from death after various brain insults has been described [102].

Whatever the precise mechanism of the effects that we have presented, the results suggest that exposure to low levels of cellular stress can reduce the vulnerability of DA neurons. This is consistent with previous studies of preconditioning in models of PD from our lab [9], [20] and others [99] and may have implications for the development of neuroprotective therapies. The ability to generalize preconditioning across disease models suggests that pharmacological activation of a protective stress response without the extracellular stress *per se* can be used to protect against neurodegeneration. On the other hand, the results also suggest that higher levels of oxidative stress – including those caused by high concentrations of amphetamines – increase the vulnerability of DA neurons. This latter result may in turn have implications for drug abuse as a risk factor for PD, a possibility that has been suggested by others [64], [103].
